# Single-Domain Antibodies and the Promise of Modular Targeting in Cancer Imaging and Treatment

**DOI:** 10.3389/fimmu.2018.00273

**Published:** 2018-02-19

**Authors:** María Elena Iezzi, Lucía Policastro, Santiago Werbajh, Osvaldo Podhajcer, Gabriela Alicia Canziani

**Affiliations:** ^1^Laboratorio de Terapia Molecular y Celular, Fundación Instituto Leloir, Instituto de Investigaciones Bioquímicas de Buenos Aires (IIBBA-CONICET), Ciudad Autónoma de Buenos Aires, Argentina; ^2^Laboratorio Nanomedicina, Gerencia de Desarrollo Tecnológico y Proyectos Especiales, Comisión Nacional de Energía Atómica, Ciudad Autónoma de Buenos Aires, Argentina

**Keywords:** camelid heavy-chain antibody, drug-like properties, bioavailability, immunogenicity, broad epitope coverage, poly-specificity

## Abstract

Monoclonal antibodies and their fragments have significantly changed the outcome of cancer in the clinic, effectively inhibiting tumor cell proliferation, triggering antibody-dependent immune effector cell activation and complement mediated cell death. Along with a continued expansion in number, diversity, and complexity of validated tumor targets there is an increasing focus on engineering recombinant antibody fragments for lead development. Single-domain antibodies (sdAbs), in particular those engineered from the variable heavy-chain fragment (VHH gene) found in Camelidae heavy-chain antibodies (or IgG2 and IgG3), are the smallest fragments that retain the full antigen-binding capacity of the antibody with advantageous properties as drugs. For similar reasons, growing attention is being paid to the yet smaller variable heavy chain new antigen receptor (VNAR) fragments found in Squalidae. sdAbs have been selected, mostly from immune VHH libraries, to inhibit or modulate enzyme activity, bind soluble factors, internalize cell membrane receptors, or block cytoplasmic targets. This succinct review is a compilation of recent data documenting the application of engineered, recombinant sdAb in the clinic as epitope recognition “modules” to build monomeric, dimeric and multimeric ligands that target, tag and stall solid tumor growth *in vivo*. Size, affinity, specificity, and the development profile of sdAbs drugs are seemingly consistent with desirable clinical efficacy and safety requirements. But the hepatotoxicity of the tetrameric anti-DR5-VHH drug in patients with pre-existing anti-drug antibodies halted the phase I clinical trial and called for a thorough pre-screening of the immune and poly-specific reactivities of the sdAb leads.

## Introduction

**Figure 1:**
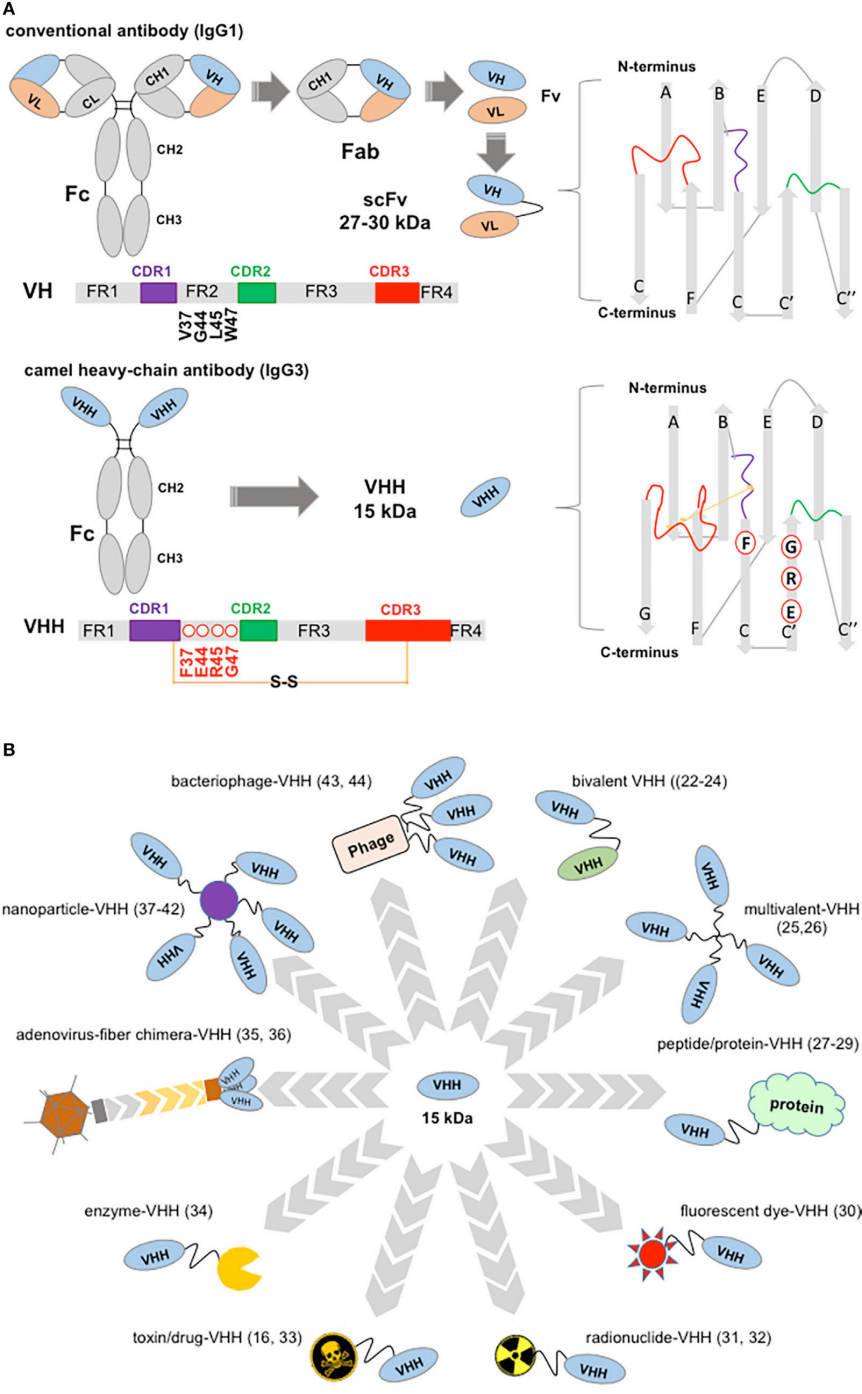
Structure of a “conventional” IgG1 and of a camelid IgG3, showing variable domain differences and illustrations of potential, VHH-based, cancer therapeutics. **(A)** Schematic of an IgG1 showing canonical hypervariable domains (left top diagram) consisting of two light (L) chains, comprising the VL and CL domains, and two heavy (H) chains composed of the VH, CH1, hinge, and CH2 and CH3 domains; and, below a camelid homodimeric heavy-chain IgG3, a heavy-chain antibody (HCAb) (left bottom diagram) which comprises only H chains; each H chain contains a short VHH hinge, CH2, and CH3 domains. The homodimeric heavy-chain IgG2 (not shown) has longer VHH hinge domains compared to IgG3 and comparable CH2, CH3. The smallest intact functional antigen-binding fragment that can be generated from the immunoglobin G (IgG) canonical variable domains, consists of an oligopeptide linked VH–VL pair known as single-chain variable fragment (top right), while the smallest intact functional antigen-binding fragment of HCAbs is the single-domain VHH (bottom right) known as Nb. VH and VHH bars show framework (FR), complementarity domain regions (CDRs) (color coded), and key residues substitutions. Non-canonical C residues are involved in an inter-CDR disulfide bond in VHH structure. **(B)** VHH-associated strategies in targeting tumors and tumor accessory cells. Top, clockwise: bivalent bi-specific VHH (22–24); multivalent, high-avidity mono-VHH molecules (25, 26); VHH fusions ranging from vascular penetration peptide-VHH to engineered hu-Fab and albumin-binding domains (27–29); fluorescent dye fusions, for example, one spontaneously crossing the blood–brain barrier (30); radionuclide-VHHs (31, 32); toxin-VHH theragnostics (16, 33); chromogenic enzyme fusions: here an alkaline phosphatase-VHH may be applied in ELISA, dot blot, and transferred protein identification in western blot (34); oncolytic virus (35, 36); VHH decorated nanoparticles for therapeutics delivery and in facilitating photothermal therapy (37–42); bacteriophage engineered to display VHH and deliver targeted therapeutics (43) may also be developed for signal amplification in ELISA assays (44).

The success of monoclonal antibodies (mAbs) in cancer therapy is driven by the overall efficacy of targeted therapies. The rate of approval of recombinant mAbs continues to outperform that of small molecules in all indications and in particular for the treatment of cancer (1, 2). However, a recent rate of advancement of antitumor candidate leads from preclinical to clinical trial was estimated to be only 20% (3). One approach to improving this success rate is to focus early on a set of characteristics termed “developability” based on high-throughput qualification tests applicable to mAb hits for a particular target. Two “developability” issues impacting candidate bioavailability are off-target binding and aggregation that can also result in toxicity and immune-reactivity. A candidate with a favorable profile is more likely to emerge from a large set of hits with a broad epitope coverage, by screening out off-target reactive mAbs (4) and guaranteeing “manufacturability,” or stability and solubility, of the lead candidate early in the pipeline (5–8). Camel and shark serum have provided a source of versatile antibody therapeutics with good “developability” and “manufacturability” prospects (6, 9–11). Most recombinant, variable heavy-chain (or VHH) single domains from homodimeric IgG2 and IgG3 found in camelids and VNAR of the so-called Ig new antigen receptor of sharks display higher solubility (above 1 mg/mL) and rapid refolding after temperature or chemical denaturation in comparison with the heterodimeric VL–VH domains in a Fab fragment (Figure 1A) (12, 13). VHH expression yield, whether in the periplasm of *Escherichia coli* or the cytoplasm of eukaryotic cells is high. Sequence identity of the VNAR domain with canonical human VH falls as low as 25%, while known camelid VHH domains are distinctly close to human VH3 germline sequences and a source of easily humanized single-domain antibody (sdAb) drugs (10, 14–16). In addition, services such as Hybribody, a platform from Hybrigenics for the selection and validation of antibodies derived from a fully synthetic humanized sdAb library displayed on phage, can supply humanized sdAbs to specific targets (Table 1, item 3) (17). The immunogenicity of humanized sdAbs may be erroneously overlooked yet it is tested in phase I clinical trials (18). The antigen-specific combining sites may be immunogenic providing sufficient justification for the early use of immunogenicity-screening platforms (19). The detection of anti-drug antibodies (ADA) using highly sensitive ELISAs at Ablynx revealed the benefit of mutating sdAb residues in hydrophobic patches at the C-terminus of VH of single-chain variable fragment (scFv) and VHH fragments, shielded by the CH domains in the original structure (20, 21).

**Table 1:** Summarized single-domain antibody (sdAb) research and development in cancer diagnostics and therapy.

Services^a^	Applied technologies	Proposed clinical benefit	Service provider^b^
1. Customizing sdAb engineering	Immune, naïve, and synthetic/humanized libraries phage display, bacterial display, intrabody library services, VHH production (45)	sdAb innovative binder formats, systems biology and target validation tools (46)	GenScript; Creative BioLabs; Lampire Biological Laboratories; Capralogics, Inc.; ProSci, Inc.; Hybrigenics Coporation, Allele Biotechnology and Pharmaceuticals, Inc.; Qoolabs, Inc.; Abcore Inc.; QVQ Holding BV; Rockland Immunochemicals, Inc.
		Pipeline construction (47)	

2. Optimizing sdAb lead candidate selection	Epitope binning and optimum epitope coverage antibodies and sdAb, tested in a pairwise combinatorial manner (8)	Multiple epitope bins reflect functional diversity, support oligoclonal therapy or the simultaneous targeting of biological pathways; watch for off-target binding (48)	Carterra, Inc.; Creative BioLabs

3. Humanizing and screening sequences to diminish sdAb immunogenicity	sdAbs humanization (15, 45) and Identification of potential immunogenic sequences (21)	lower sdAb immunogenicity	GlobalBio, Inc.; Creative BioLabs; Hybrigenics Coporation; EpiVax, Inc.

4. Tailoring the sdAb *in vivo* half-life	Half-life optimization in circulation (49); Nanobody^®^-based half-life extension technology	Ozoralizumab, a next-generation bivalent tumor necrosis factor alpha (TNFα) blocker linked to a low-affinity albumin-binding domain	Ablynx; Eddingpharm

**Applications^c^**	**Targeted tumor antigens**	**Clinical trials**	**Developer^b^**

5. Overcoming monoclonal antibody limitations by targeting inaccessible and intracellular tumor antigens	CapG (50), non-endocytic co-transport and cytoplasmic translocation (51), DR5 (52), dynamic transformation (53), Glioblastoma (54), CA9/CAIX activity (55), p53–HDM2 disruption (56), mesothelin (57)	not initiated or halted	Novartis; ProSci Inc.; Hybrigenics Services; QVQ Holding BV

6. Selecting proficient probes for molecular imaging	^131^I-SGMIB Anti-HER2 sdAb	Phase I, CAM-VHH1 Study NCT02683083	Camel-IDS NV, TBM program^d^ (social, non-profit organization), QVQ holding BV
	^68^Ga-HER2-sdAb (near infrared) probes in sentinel lymph node detection or residual tumor tissue (58)	Phase II PET/CT. Clinical Trial II	

7. Targeting known tumor antigens	Epithelial growth factor receptor (59), carcinoembryonic antigen (60), prostate-specific membrane antigen, anti-VEGF/Ang2 (BI 836880 Nb^®^), anti-RANKL (ALX-0141 Nb^®^), TNFα, ADAMTS5	Phase I, Boehringer Ingelheim, anti-VEGF/Ang2 Nb^®^, safety in cancer patients	Ablynx/Merk; Boehringer Ingelheim; Eddingpharm, clinical development, registration and commercialization in Greater China of anti-RANKL Nb^®^ and ozoralizumab; Merk KGaA
		Phase I, Ablynx (ALX-0141 Nb^®^) safety and pharmacokinetic study	
		Anti-ADAMTS5, M6495 Nb^®^ Interventional, Merk KGaG in healthy volunteers. NCT03224702	

8. Targeting immune checkpoints	PD-L1 (61), CD47/SIRP α axis (62, 63), glucocorticoid-induced TNFR-related protein	Early Phase I, ^99m^Tc labeled anti-PD-L1 sdAb for diagnostic imaging of non-small cell lung cancer. Pending. NCT02978196	Merck & Co.; Merck KGaA; Ablynx

9. Testing molecular mimicry, including anti-idiotypes and abzymes	Ab2 abzymes with alliinase activities (64), self-diversifying antibody library platform (SDALib)	New drug diacovery using Abzyme’s yeast-based camelid single domain VHH antibody library with self-diversifying ability, to generate VHH antibodies against cancer-related target isoforms	Abzyme Therapeutics, LLC and Ibex BioSciences, LLC partnership

*^a^Services that support sdAb generation and lead candidates screening*.

*^b^Search business firm information with preferred online engine*.

*^c^Applications that may broaden the range of tumor targeting lead candidate*.

*^d^http://www.innovatienetwerk.be/projects/2275*.

The VHH repertoire is as complex in sequence diversity as is the IgG1 VH camelid counterpart (65–67). Total peripheral blood lymphocytes and lymph node ribonucleic acid (RNA) from alpaca, llamas, dromedaries, and camels are easily extracted to build recombinant VHH libraries. Typically, a VHH phage display library containing 6 × 10^7^ VHHs clones are generated from 200 µg processed RNA and diverse polymerase chain reaction strategies are available to amplify VHH gene fragments from lymphocyte complementary deoxyribonucleic acid (68, 69). Several reports have confirmed the ease of engineering sdAbs (69, 70) and of selecting specific binders against conformational epitopes in comparison with hit selection of scFv, where library construction shuffles their immune specificity (68, 71, 72).

Two or three VHHs have been combined in a single polypeptide chain to express single, dual, or multimeric specificities without compromising folding or the binding affinities (22, 73). In addition, “self-associating peptide” constructs have been designed to match VHH pairs (69, 74). Concomitantly, the experience gained in site-specific conjugations, in particular those driven by targeted enzymatic reactions, has ensured the preservation of antigen-binding properties of sdAbs (31, 75). The reported affinities of VHH fragments fall in the nanomolar to picomolar range, with binding kinetics comparable to those of conventional antibodies. Selection of stable antigen complexes is often the result of applying selection pressures, such as stringent washing, that enrich a library in VHH with slower off-rates while competitive elution was reported in selecting fragments with novel epitope targeting (70, 76–79). VHH genes are an established source of antibodies, as evidenced by the number of reported co-crystal structures (68, 80–82). Figure 1A highlights hallmark VHH residues and, when present, an inter-CDR disulfide bond in the VHH sequence. Around 10% of HcAbs lack these hydrophobic residues mutation but often show longer CDR3 covering putative VL contacts or a hydrophilic substitution of Trp118. Gonzalez-Sapienza et al. suggested a plausible mechanism of selection of HcAb producing B-cells that supports the emergence of independently folding, soluble VH and VHH domains (72).

## Distinctive Properties of sdAbs

The ease of selecting sdAb under denaturing conditions has assisted in the isolation of “superstable” species with improved resistance to proteases that were proposed as antimicrobial therapeutics of oral intake (83, 84). Li et al. have successfully selected VHH expression products with a high isoelectric point (pI) that spontaneously crossed the blood–brain barrier (transcytosis) (30). High-pI sdAb have been found to penetrate cells and bind to intracellular proteins. For instance, a sdAb that bound specifically to the hepatitis C virus (HCV) protease, selected for its ability to penetrate cells (transbodies), interfered with heterologous HCV replication (15). A sdAb-based anti-β-catenin intrabody was expressed and folded in the cytoplasm retaining its ability to bind to β-catenin (85).

The solvent accessible surface (SAS) area of antigen-VHH and VNAR complexes are comparable to antigen–VH–VL complex SAS indicating that complementarity domain region (CDR) loops involved in antigen binding (Figure 1A) contribute similar surface contacts. VHH H1 and H3 loops connecting the β-sheets of the VHH domain are flexible, sometimes longer and packed in a less compact fashion compared to canonical VH of murine and human immunoglobin G (IgGs) (10, 86). Co-crystal structures of enzyme-VHH and -VNAR complexes showed CDRs that often protruded into the active-site cleft and the derived sdAbs were later shown to inhibit catalysis (65, 66, 87, 88). Alternatively, sdAbs have been selected to stabilize “drugable” targets that display multiple conformations (or conformational plasticity) (79, 82). For example, the urokinase-type plasminogen activator (uPA) from the trypsin-like serine protease family, a target involved in metastasis, is known to adopt high and low activity conformations. Selection of sdAbs against mouse uPA yielded both a catalytic-site inhibitor and an allosteric ligand. Crystal structures of the uPA sdAb complexes revealed high and low activity determinants that provided clues of therapeutic value on the regulatory determinants of uPA and of trypsin-like serine proteases in general (89). Table 1 documents the pharmaceutical relevance of sdAbs through the number of research and development companies involved in novel sdAb generation, available contract services, lead candidates under clinical trial, and examples of the sdAbs more recently generated against cancer targets.

## sdAbs in Imaging Applications for Cancer Diagnostics

Molecular imaging techniques, of widespread use in the clinic, allow the non-invasive quantitation and visualization of tumors *in vivo* and sdAbs have become promising, small-sized, high-affinity tracers (58, 90–92) (Figure 1B). Nuclear imaging probes associated to sdAbs have been evaluated in both single-photon emission computed tomography (SPECT) and positron emission tomography (PET) (90, 93) (Table 1, item 6). The most advanced sdAb under clinical evaluation is the ^68^Ga-labeled anti-HER2 sdAb 2Rs15d probe, developed to screen candidates who qualify for treatment with an anti-HER2 therapeutics. A phase I study resulted in high-quality images without adverse reactions and retained 10% of injected activity in blood after 1 h (94). A phase II trial was launched to correlate tumor uptake with HER2 levels in biopsies of 160 metastatic breast carcinoma patients (Table 1, item 6). In other studies, 2Rs15d labeled with the prosthetic group *N*-succinimidyl-4-[^18^F] fluorobenzoate ([^18^F]-SFB) was validated in preclinical models to advance PET imaging (95). The specific uptake of the sdAb 2Rs15d probe in HER2-positive tumor xenografts showed high tumor-to-blood and tumor-to-muscle ratios, high contrast PET imaging and fast renal clearance (4% intra auricular/g at 3 h post injection.). The lead candidate MSB0010853, a biparatopic sdAb labeled with ^89^Zr bound efficiently to HER3 kinase, a potential clinical target associated with resistance to epithelial growth factor receptor (EGFR) and HER2 targeted therapies (96, 97).

Organometallic radiopharmaceuticals are also widely used in diagnosis with SPECT imaging. sdAbs that target either EGFR (98), VCAM1, an 8-kDa fragment of gelsolin or carcinoembryonic antigen (CEA) have been conjugated with ^99m^Tc (99). Recently, an anti-PD-L1 sdAb labeled with ^99m^Tc discriminated wild type mice from PD-L1 knock-out mice by SPECT/CT imaging (100). sdAbs used as fluorescence-guided near-infrared wavelength range (NIR) probes are also under preclinical studies addressing sentinel lymph node imaging quality and guiding surgical/endoscopic removal of residual tumor tissue (101). NIR probes, IRDye800CW or IRDye680RD, were conjugated either by lysines or C-terminal cysteine to the 7D12 anti-EGFR sdAb. After IR dye conjugation, comparable specificities and affinities of 7D12 and the conjugate were measured toward EGFR *in vitro* (58, 102). This study also showed an accumulation of the cysteine-conjugated 7D12 in A431 human tumor xenografts in nude mice or high tumor-to-muscle ratio.

The ultrasound imaging of vessel cell adhesion protein 1 (VCAM1), using specific sdAbs coupled to lipid microbubbles as contrast enhancers, is used to assess potential adhesion sites of melanoma cell extravasation and metastasis (75). Although sdAbs are promising imaging probes renal retention during clearance and toxicity were reported in preclinical studies. Adverse effects were attributed to the polar residue number favoring the interaction with the megalin/cubilin system in the renal tubuli (103). This issue was overcome by mutating positive residues, facilitating filtration at the negatively charged glomerular membrane (104). Toxicity was also controlled by gelofusine or lysine added to the probe (103, 105).

## sdAb Against Tumor Targets for Clinical Use

Single-domain antibodies that bind either hepatocyte growth factor, EGFR, bone morphogenetic protein (TGFb superfamily growth factors), HER2, cMET, or VEGFR1, have been shown to efficiently block tumor cell proliferation (81, 106–109). Zhang et al. (61) have recently shown that KN035, an anti-PD-L1 sdAb, can induce T-cell responses and inhibit tumor growth; the KN035 CDRs structure is remarkably similar to that of the VH of Federal Drug Administration-approved Durvalumab (110). Other sdAbs were developed to target uPA, and chemokine receptors such as CXCR4 and CXCR7 (111). More recently, sdAbs targeting antioxidant enzymes such as membrane catalase and superoxide dismutase were selected for their ability to induce reactive oxygen species-dependent cancer cell apoptosis and found to be synergetic to chemotherapy (112).

Single-domain antibodies modules have been engineered into multivalent structures to overcome fast clearance. The anti-DR5 sdAb tetramer showed excellent pharmacokinetics and efficacy in preclinical models, inducing robust antitumor responses and sustained caspase activation *in vivo*. However, in the phase I trial an unexpected hepatotoxicity which triggered hepatocyte apoptosis, later associated to the immune crosslinking of the tetramer in those patients with pre-existing ADA, prompted its discontinuation (113). A bifunctional sdAb, targeting EGFR and TRAIL, inhibits the growth of different tumor cell types that were not responsive to either EGFR-antagonist or death receptor-agonist monotherapies is a clear step forward of the clinical application of sdAb modules (23). To improve the efficacy of a bifunctional therapeutic, the MaAbNA-PEG2000-ADM chimera consisting of an anti-EGFR1 sdAb linked to two anti-HER2 affibodies was conjugated with Adriamycin (114). The bispecific sdAb chimera recognizing CEA and antigen cluster of differentiation 16 (CD16) (NK-cell marker) was linked to a mutated human IgG1 Fc-fragment that equipped the dimer with an effector function (115). The bispecific antibody HER2-S-Fab, an anti-CD16 sdAb that is linked to a anti-trastuzumab Fab, also exhibited a potent tumor growth inhibition in a human tumor xenografts model (29). A multivalent, sdAb-based, in-tandem trimer was capable of simultaneously binding to CEA, EGFR, and green fluorescence protein with high efficacy for inhibition of human epidermoid carcinoma A431 cell proliferation (26). An interesting approach to increase the half-life of sdAbs without affecting the affinity for its target was the fusion between an anti-TNFα sdAb with an albumin-binding domain derived from *Streptococcus zooepidemicus* (~39-fold half-life increase with respect to the sdAb alone, Table 1, item 4) (28).

Targeting tumors with ionizing radiation is also a promising area for growth for sdAb therapeutics. The most relevant *in vivo* study demonstrated that i.v. administration of the sdAb anti-HER2 labeled with ^177^Lu, a γ-emission radionuclide, completely prevented tumor growth in mice with small HER2-positive tumors (32). The α-emitting radionuclides ^213^Bi and ^211^At coupled to sdAbs are tentatively used to treat minimal residual disease and micro-metastasis and their clinical application is being intensely explored (116).

## Emerging Drug-Delivery Strategies That Use sdAbs

To improve solid tumor penetration an EGFR-targeted sdAb was fused to an iRGD, a cyclic domain selective of αvβ3 and αvβ5 integrins that carries a CendR motif that binds neuropilin 1 (NRP-1) (117). The efficacy of this construct was measured in BGC-823 multicellular spheroids that overexpress EGFR, NRP-1, and integrins. The anti-EGFRsdAb-iRGD showed better performance in reducing spheroid size than anti-EGFRsdAb or cetuximab. *In vivo*, anti-EGFRsdAb-iRGD-FITC was shown to bind to αvβ3 and αvβ5 expressed in the tumor vessels, malignant cells, and cancer-associated stromal cells, penetrating further than the anti-EGFR-FITC (27). Recently, anti-EGFRsdAb-iRGD was conjugated to silk fibroin nanoparticles loaded with paclitaxel, resulting in a significant anti-neoplastic activity in EGFR-expressing cells *in vitro* and *in vivo* (41).

Single-domain antibody has been successfully used to retarget oncolytic adenovirus to a non-cognate receptor following the incorporation of an anti-CEA sdAb into the adenovirus capsid fiber (Figure 1B). This modification was shown to control viral tropism, entry, and gene transfer specifically in CEA-overexpressing cells (36, 118). sdAb displayed on genetically engineering phage combined with target drugs or imaging probes has recently been proposed for preclinical evaluation (43, 119).

Single-domain antibodies have been used to retarget nanoparticles with particular diagnostic or therapeutic properties (120, 121). Branched gold nanoparticles functionalized with an anti-prostate-specific antigen sdAb were shown to destroy cancer cells in response to laser irradiation in a preclinical model of photothermal therapy (37). Pegylated liposomes, schematized in Figure 1B, may be re-directed away from the reticuloenthoelial system by coupled sdAbs and are under preclinical evaluation as drug nanocarriers (39, 40). A novel potent delivery system based on extracellular vesicles (EVs) has recently been described where an anti-EGFR sdAb was anchored on the surface of EVs *via* glycosylphosphatidylinositol signal peptides derived from the decay-accelerating factor significantly improving EV targeting (42).

## Platforms for the Generation of New sdAbs

Epitope recognition and coverage appear to be dependent on immune-selection pressure of VH and VHH sequences *in vivo* and by the library diversity (122, 123). To amplify antigenic epitope coverage, naïve and semi-synthetic libraries are being promoted to amplify antigen epitope coverage often limited by B-cell IgG amplification *in vivo*. Low affinities may be matured or optimized as required. sdAb discovery may now count on high-throughput, high-resolution broad epitope coverage analysis and poly-specificity and affinity screening tools to increase the likelihood of selecting sdAbs with the desired therapeutic functions (Table 1, item 2) as well as to discriminate between functional sdAbs, such as those that can trigger receptor internalization (124) and polyreactive leads (8).

Three novel VHH library presentation and selection platforms have been recently proposed for a high-throughput selection of sdAb to integral membrane tumor antigens, or proteins overexpressed on the surface of whole cells or on virus-like particles (70, 123). Two of the platforms were designed to identify binders to antigen diluted in lysates or in complex mixtures for the discovery of sdAbs that bind critical pathway targets (78, 125). Rosotti et al. reported high throughput, parallel selection and characterization strategies to identify phage-displayed sdAbs against receptors expressed on murine bone marrow-derived dendritic cells (123). As a result of *en masse* cloning and whole-cell screening, the *in vivo* biotinylation of selected VHH facilitated the identification of targets. The isolated VHH were effectively mapped, or binned, by epitope, and target coverage was recorded [also see Ref. (126), Table 1, item 2].

Salema and Fernandez optimized the display of VHH on Gram-negative *E. coli*, and the direct expression of selected VHH clones, by anchoring the expression product on the outer membrane by fusing to the N-terminal, intimin β-domain (Neae) (78, 127, 128). High-affinity clone selection was optimized by magnetic cell sorting on immobilized recombinant biotinylated antigen (MACS) or by flow cytometry on whole cells (CellS) (78).

A third sdAb selection platform was presented by Cavallari using a Gram-positive Staphylococcal (*Staphylococcus aureus*) display of sdAb (125). Here, VHH clones were engineered with the signal peptide from staphylococcal enterotoxin B, with the sortase A (SrtA) LPXTG motif, to display folded VHH on the surface. Endogenous SrtA covalently, and irreversibly, coupled expressed sdAb on the outer membrane. A nucleophilic attack of the SrtA sdAb-acyl intermediate by polyglycine nucleophile-biotin was used to release and biotinylate selected VHH clones. The major advantages of bacterial display were the efficiency of selection as reflected by a high “hit” frequency, or high frequency of success, in comparison to hit selection by phage display, and minimum avidity. Also attractive is the choice of evaluating selected sdAbs by flow cytometry or in SPR binding assays directly enabling screening sdAbs by epitope and a discrimination of poly-specificity in a high-throughput mode (78, 128).

## Concluding Remarks

Single-domain antibodies are soluble, stable, recombinant proteins that fold independently and display an outstanding versatility. The hardware-building concept of “plug and play” appears as an excellent paradigm in which sdAbs are part of a therapeutics generation tool kit that includes engineered recombinant sdAbs, radionuclides, dyes, peptides, proteins, nanostructures, phage, and virus.

Currently, 20–25% of the mAbs in clinical development for cancer and non-cancer indications are recombinant human antibodies derived from phage display libraries or from transgenic mice. Five antibody “fragments” (scFv) were reported in clinical phase 2/3 this past year. These include a human scFv-doxorubicin loaded liposome; two scFv conjugates, a humanized anti-EpCAM scFv-immunotoxin conjugate; and an anti-fibronectin extra-domain B human scFv for cancer indications.

The unexpected toxicity of the anti-DR5 tetramer, TAS266, opened the question of pre-existing immunity against sdAb. This issue has been addressed by developing sensitive immune serum assays and immunogenicity-screening platforms (Table 1, item 3, EpiVax) to identify the safer lead candidates, helping reduce the risk of clinical trial failure of sdAb-based drugs. The promise of recombinant, engineered, antibody-based building modules with optimal efficacy and biovailability may soon translate into tangible cancer drugs.

## Author Contributions

MI, LP, SW, OP, and GC: conception, design, and writing of the review manuscript.

## Conflict of Interest Statement

The authors declare that the research was conducted in the absence of any commercial or financial relationships that could be construed as a potential conflict of interest.

## Abbreviations

A431, epidermoid carcinoma cell line; Abzyme, an antibody with catalytic activity, binding a chemical group and stabilizing the transition state of a given reaction; ADA, anti-drug antibody; ADAMTS5, a disintegrin and metalloproteinase with thrombospondin motifs; ADM, Adriamycin; BMCD, bone marrow culture-derived macrophages; BMP, bone morphogenetic protein; CA9/CAIX, carbonic anhydrase IX encoded by the CA9 gene; CD47/SIRP α axis, cluster of differentiation 47 and the myeloid inhibitory immunoreceptor signal regulatory protein α signaling axis; CapG, macrophage capping protein oncogene; CD16, cluster of differentiation 16, a low-affinity Fc receptor; CDR, complementarity determining region or antigen-binding domain; CendR, C-end rule motif R/KXXR/K; CEA, carcinoembryonic antigen; cDNA, complementary deoxyribonucleic acid; cMET, tyrosine-protein kinase Met or hepatocyte growth factor receptor (HGFR); CXCR4, fusin or CD184; CXCR7, atypical chemokine receptor 3 or GPCR 159; DAF, decay-accelerating factor; DR5, death receptor 5 of the TNF receptor superfamily (TNFRSF) 5; EGFR, epidermal growth factos receptor, a membrane tyrosine kinase; EpCAM, epithelial cell adhesion molecule or TROP1; EV, extracellular vesicle; Fab, immunoglobulin antigen-binding fragment composed of one constant and one variable domain of each of the heavy and the light chain; Fc, fragment crystallizable region of Ig; FDA, Federal Drug Administration; FR, framework region is a subdivision of the mAb variable region; FTCI, fluorescein isothiocyanate; GFP, green fluorescence protein; GITR, glucocorticoid-induced TNFR-related protein; GPI-DAF, glycosylphosphatidylinositol-anchored decay-accelerating factor; HcAbs, heavy-chain antibodies; HCV, hepatitis C virus; HER2, human epidermal growth factor receptor 2/neu tyrosine kinase, erbB-2; HER3, human epidermal growth factor receptor tyrosine-protein kinase erbB-3; HGF, hepatocyte growth factor; IA, intra auricular; IgG, immunoglobin G; IgNAR, Ig new antigen receptor; IR, infrared; 131I-SGMIB, iodine-131- labelled N-succinimidyl 4-guanidinomethyl-3-iodobenzoate; iRGF, 9-amino acid cyclic peptide (sequence: CRGDKGPDC) binding tumor cells; i.v., intra venous; mAb, monoclonal antibody; Neae, N-terminal fragment of enterohemorrhagic *E. coli* intimin; NIR, near infrared; NRP-1, neuropilin 1; PBL, peripheral blood lymphocytes; PCR, polymerase chain reaction; PEG2000, poly(ethylene glycol) methyl ether, average Mw 2,000; PD-L1, Programmed death-ligand 1, CD274; p.i., post injection; pI, isoelectric point; PSA, Prostate-specific antigen; PSMA, prostate-specific membrane antigen; p53-HDM2, functional p53 and human double minute 2 interaction; SAS, solvent accessible surface; scFv, Single-chain variable fragment; sdAb, single-domain antibody fragment; SPECT, single-photon emission computed tomography; SPECT/CT, image fusion for anatomic imaging (CT or MRI) and functional imaging (SPECT) computed tomographies; SrtA, sortase A; RANKL, receptor activator of nuclear factor kappa-B ligand; RNA, ribonucleic acid; TNFα, tumor necrosis factor alpha; uPA, urokinase-type plasminogen activator; VCAM1, vascular cell adhesion protein 1; VEGF/Ang2, vascular endothelial factor/angiopoietin-2; VEGFR1, vascular endothelial growth factor receptor 1; VHH, heavy-chain only antibody fragment or nanobody; VH and VL, variable heavy and light chain domains from conventional IgG structures; VNAR, variable new antigen receptor single domain.
